# 4-[Bis(1*H*-indol-3-yl)meth­yl]benzonitrile

**DOI:** 10.1107/S1600536811020411

**Published:** 2011-06-11

**Authors:** Xiang Deng, Di Wu, Xiaomei Huang, Feihua Luo

**Affiliations:** a Department of Chemistry and Chemical Engineering, Sichuan University of Arts and Science, Sichuan Key Laboratory of Characteristic Plant Development Research, Sichuan Dazhou 635000, People’s Republic of China

## Abstract

In the title mol­ecule, C_24_H_17_N_3_, the didhedral angles formed by the mean planes of the indole ring systems and the benzene ring are 86.44 (7) and 86.96 (7)°. The dihedral angle between the two indole ring systems is 72.08 (6)°. In the crystal, inter­molecular bifurcated (N—H)_2_⋯N hydrogen bonds link mol­ecules into sheets lying parallel to (010).

## Related literature

For background and the biological activity of bis­indolylalkanes and their derivatives, see: Bell *et al.* (1994 ▶). For related structures, see: Govindasamy *et al.* (1998 ▶); Krishna, Velmurugan, Babu & Perumal (1999 ▶); Krishna, Velmurugan & Shanmuga Sundara (1999 ▶); Seetharaman & Rajan (1995 ▶). For standard bond-length data, see: Allen *et al.* (1987 ▶).
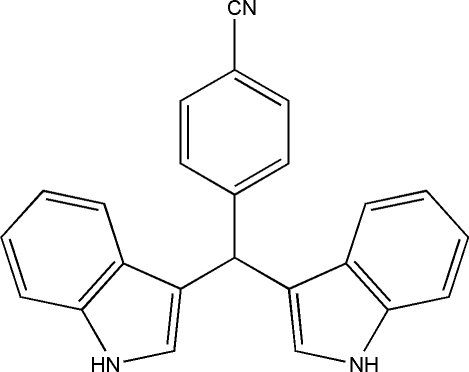


## Experimental

### 

#### Crystal data

C_24_H_17_N_3_
*M* *_r_* = 347.41
Monoclinic, 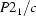

*a* = 9.5882 (12) Å
*b* = 19.155 (3) Å
*c* = 10.3801 (13) Å
β = 100.562 (3)°
*V* = 1874.1 (4) Å^3^
*Z* = 4
Mo *K*α radiation
μ = 0.07 mm^−1^
*T* = 296 K
0.20 × 0.15 × 0.09 mm

#### Data collection

Bruker SMART CCD diffractometer
14081 measured reflections
3292 independent reflections
2613 reflections with *I* > 2σ(*I*)
*R*_int_ = 0.036

#### Refinement

*R*[*F* ^2^ > 2σ(*F* ^2^)] = 0.041
*wR*(*F* ^2^) = 0.110
*S* = 1.05
3292 reflections
253 parameters
H atoms treated by a mixture of independent and constrained refinement
Δρ_max_ = 0.15 e Å^−3^
Δρ_min_ = −0.13 e Å^−3^

### 

Data collection: *SMART* (Bruker, 2007 ▶); cell refinement: *SAINT* (Bruker, 2007 ▶); data reduction: *SAINT*; program(s) used to solve structure: *SHELXS97* (Sheldrick, 2008 ▶); program(s) used to refine structure: *SHELXL97* (Sheldrick, 2008 ▶); molecular graphics: *PLATON* (Spek, 2009 ▶); software used to prepare material for publication: *SHELXTL* (Sheldrick, 2008 ▶).

## Supplementary Material

Crystal structure: contains datablock(s) I, global. DOI: 10.1107/S1600536811020411/lh5252sup1.cif

Structure factors: contains datablock(s) I. DOI: 10.1107/S1600536811020411/lh5252Isup2.hkl

Supplementary material file. DOI: 10.1107/S1600536811020411/lh5252Isup3.cml

Additional supplementary materials:  crystallographic information; 3D view; checkCIF report

## Figures and Tables

**Table 1:** Hydrogen-bond geometry (Å, °)

*D*—H⋯*A*	*D*—H	H⋯*A*	*D*⋯*A*	*D*—H⋯*A*
N3—H3*A*⋯N1^i^	0.86 (2)	2.22 (2)	3.084 (2)	178.6 (18)
N2—H2*A*⋯N1^ii^	0.91 (2)	2.34 (2)	3.206 (2)	160.3 (19)

Symmetry codes: (i) 
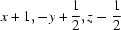
; (ii) 
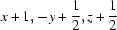
.

**Fig. 1.**
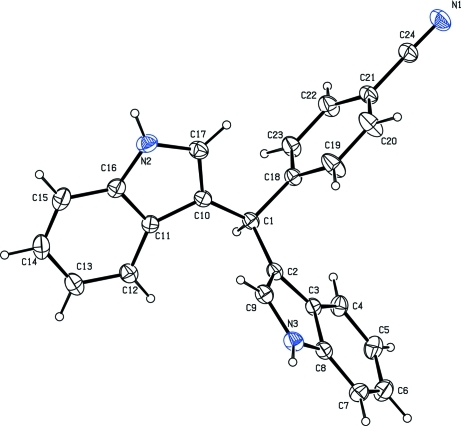
The molecular structure of the title compound with 30% probability ellipsoids.

**Fig. 2.**
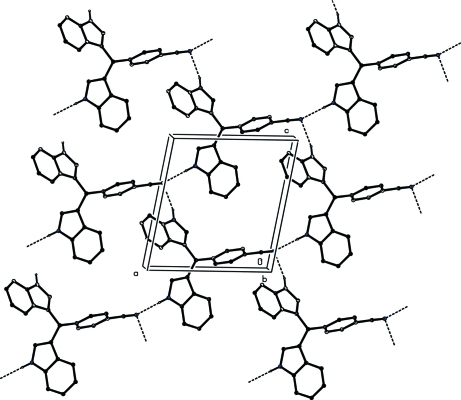
Part of the crystal structure with hydrogen bonds shown as dashed lines. Only H atoms involved in the hydrogen bonds are shown.

## supplementary crystallographic information

### Comment

Bisindolylalkanes and their derivatives constitute an important group of bioactive metabolites of terrestrial and marine origin (Bell *et al.*, 1994). We report here the crystal structure of the title compound (I). In the molecular structure, (Fig. 1) the bond lengths (Allen *et al.*, 1987) and and angles are within normal ranges and those in the indole group are in agreement with related structures (Govindasamy *et al.*, 1998; Krishna, Velmurugan, Babu & Perumal, 1999; Krishna, Velmurugan & Shanmuga Sundara, 1999; Seetharaman & Rajan, 1995). The didhedral angles formed by the mean planes of the indole ring systems and the benzene ring are 86.44 (7) and 86.96 (7)°. The dihedral angle between the two indole ring systems is 72.08 (6)°. In the crystal, intermolecular bifurcated (N—H)x2···N hydrogen bonds link molecules into two-dimensional sheets parallel to (010) (Fig. 2).

### Experimental

A mixuture of 4-cyanobenzaldehyde (1 mmol), indole (2 mmol) and I_2_ (0.2 mmol) in acetonitrile (10 ml) was stirred at room temperature for a few s. After completion of the reaction, the mixture treated with aq. Na_2_S_2_O_3_ solution (5%, 10 ml) and the product was extracted with ethyl acetate (3×5 ml). The combined organic layer was dried with anhydrous sodium sulfate, concentrated *in vacuo* and purified by column chromatography (ethyl acetate: petroleum ether=1:9) to afford the pure product. Crystals of (I) suitable for X-ray diffraction were obtained by slow evaporation of a methanol solution.

### Refinement

H atoms bonded to C atoms were placed in calculated positions and refined in a riding-model approximation with C—H = 0.93 Å and *U*_iso_(H)= 1.2Ueq(C). The H atoms bonded to N atoms were refined independently with isotropic displacement parameters.

### Crystal data

**Table:**

C_24_H_17_N_3_	*F*(000) = 728
*M**_r_* = 347.41	*D*_x_ = 1.231 Mg m^−^^3^
Monoclinic, *P*2_1_/*c*	Mo *K*α radiation, λ = 0.71073 Å
Hall symbol: -P 2ybc	Cell parameters from 3543 reflections
*a* = 9.5882 (12) Å	θ = 2.9–24.6°
*b* = 19.155 (3) Å	µ = 0.07 mm^−^^1^
*c* = 10.3801 (13) Å	*T* = 296 K
β = 100.562 (3)°	Block, colourless
*V* = 1874.1 (4) Å^3^	0.20 × 0.15 × 0.09 mm
*Z* = 4	

### Data collection

**Table:**

Bruker SMART CCD diffractometer	2613 reflections with *I* > 2σ(*I*)
Radiation source: fine-focus sealed tube	*R*_int_ = 0.036
graphite	θ_max_ = 25.0°, θ_min_ = 2.1°
φ and ω scans	*h* = −11→11
14081 measured reflections	*k* = −22→22
3292 independent reflections	*l* = −12→12

### Refinement

**Table:**

Refinement on *F*^2^	Secondary atom site location: difference Fourier map
Least-squares matrix: full	Hydrogen site location: inferred from neighbouring sites
*R*[*F*^2^ > 2σ(*F*^2^)] = 0.041	H atoms treated by a mixture of independent and constrained refinement
*wR*(*F*^2^) = 0.110	*w* = 1/[σ^2^(*F*_o_^2^) + (0.0494*P*)^2^ + 0.3483*P*] where *P* = (*F*_o_^2^ + 2*F*_c_^2^)/3
*S* = 1.05	(Δ/σ)_max_ < 0.001
3292 reflections	Δρ_max_ = 0.15 e Å^−^^3^
253 parameters	Δρ_min_ = −0.13 e Å^−^^3^
0 restraints	Extinction correction: *SHELXL97* (Sheldrick, 2008), Fc^*^=kFc[1+0.001xFc^2^λ^3^/sin(2θ)]^-1/4^
Primary atom site location: structure-invariant direct methods	Extinction coefficient: 0.030 (2)

### Special details

**Table:**

Geometry. All e.s.d.'s (except the e.s.d. in the dihedral angle between two l.s. planes) are estimated using the full covariance matrix. The cell e.s.d.'s are taken into account individually in the estimation of e.s.d.'s in distances, angles and torsion angles; correlations between e.s.d.'s in cell parameters are only used when they are defined by crystal symmetry. An approximate (isotropic) treatment of cell e.s.d.'s is used for estimating e.s.d.'s involving l.s. planes.
Refinement. Refinement of *F*^2^ against ALL reflections. The weighted *R*-factor *wR* and goodness of fit *S* are based on *F*^2^, conventional *R*-factors *R* are based on *F*, with *F* set to zero for negative *F*^2^. The threshold expression of *F*^2^ > σ(*F*^2^) is used only for calculating *R*-factors(gt) *etc*. and is not relevant to the choice of reflections for refinement. *R*-factors based on *F*^2^ are statistically about twice as large as those based on *F*, and *R*- factors based on ALL data will be even larger.

### Fractional atomic coordinates and isotropic or equivalent isotropic displacement parameters (Å^2^)

**Table:**

	*x*	*y*	*z*	*U*_iso_*/*U*_eq_	
C1	0.58636 (15)	0.10803 (8)	0.05439 (14)	0.0403 (4)	
H1	0.5637	0.0581	0.0542	0.048*	
C2	0.62867 (15)	0.12277 (7)	−0.07541 (15)	0.0403 (4)	
C3	0.54762 (15)	0.10350 (8)	−0.20061 (15)	0.0421 (4)	
C4	0.41722 (17)	0.06995 (9)	−0.23996 (16)	0.0520 (4)	
H4	0.3633	0.0566	−0.1782	0.062*	
C5	0.3700 (2)	0.05705 (10)	−0.37001 (18)	0.0652 (5)	
H5	0.2839	0.0342	−0.3963	0.078*	
C6	0.4482 (2)	0.07750 (11)	−0.46368 (19)	0.0690 (5)	
H6	0.4132	0.0685	−0.5517	0.083*	
C7	0.5762 (2)	0.11079 (10)	−0.42865 (17)	0.0627 (5)	
H7	0.6286	0.1242	−0.4915	0.075*	
C8	0.62532 (16)	0.12383 (8)	−0.29631 (16)	0.0467 (4)	
C9	0.74842 (16)	0.15361 (8)	−0.10002 (17)	0.0477 (4)	
H9	0.8208	0.1714	−0.0365	0.057*	
C10	0.70384 (15)	0.11951 (8)	0.16965 (15)	0.0419 (4)	
C11	0.82977 (15)	0.07764 (8)	0.20221 (15)	0.0425 (4)	
C12	0.88333 (17)	0.02088 (9)	0.14286 (17)	0.0526 (4)	
H12	0.8366	0.0042	0.0623	0.063*	
C13	1.00687 (19)	−0.01001 (10)	0.2058 (2)	0.0657 (5)	
H13	1.0432	−0.0481	0.1674	0.079*	
C14	1.0784 (2)	0.01494 (11)	0.3263 (2)	0.0686 (5)	
H14	1.1610	−0.0073	0.3671	0.082*	
C15	1.02992 (19)	0.07111 (11)	0.38510 (18)	0.0624 (5)	
H15	1.0783	0.0878	0.4650	0.075*	
C16	0.90608 (16)	0.10251 (9)	0.32187 (15)	0.0496 (4)	
C17	0.71121 (17)	0.16730 (9)	0.26731 (16)	0.0517 (4)	
H17	0.6434	0.2015	0.2715	0.062*	
C18	0.45208 (15)	0.14628 (8)	0.07105 (15)	0.0422 (4)	
C19	0.42852 (19)	0.21445 (10)	0.0326 (2)	0.0742 (6)	
H19	0.4949	0.2373	−0.0072	0.089*	
C20	0.3091 (2)	0.24975 (10)	0.0516 (2)	0.0786 (7)	
H20	0.2961	0.2962	0.0262	0.094*	
C21	0.20928 (16)	0.21613 (9)	0.10831 (17)	0.0528 (4)	
C22	0.23052 (18)	0.14779 (10)	0.14648 (19)	0.0647 (5)	
H22	0.1631	0.1246	0.1846	0.078*	
C23	0.35136 (17)	0.11352 (9)	0.12836 (17)	0.0569 (5)	
H23	0.3653	0.0673	0.1554	0.068*	
C24	0.08275 (18)	0.25223 (10)	0.12602 (18)	0.0602 (5)	
N1	−0.01856 (16)	0.28075 (10)	0.13748 (17)	0.0757 (5)	
N2	0.83216 (15)	0.15817 (8)	0.35857 (15)	0.0585 (4)	
H2A	0.856 (2)	0.1831 (12)	0.434 (2)	0.088 (7)*	
N3	0.74724 (15)	0.15470 (7)	−0.23225 (15)	0.0539 (4)	
H3A	0.814 (2)	0.1722 (10)	−0.2680 (19)	0.069 (6)*	

### Atomic displacement parameters (Å^2^)

**Table:**

	*U*^11^	*U*^22^	*U*^33^	*U*^12^	*U*^13^	*U*^23^
C1	0.0363 (8)	0.0372 (8)	0.0485 (9)	0.0003 (6)	0.0109 (7)	0.0017 (7)
C2	0.0330 (7)	0.0403 (8)	0.0488 (9)	0.0030 (6)	0.0102 (7)	0.0026 (7)
C3	0.0387 (8)	0.0405 (8)	0.0478 (9)	0.0061 (7)	0.0101 (7)	0.0065 (7)
C4	0.0448 (9)	0.0555 (10)	0.0540 (10)	−0.0033 (8)	0.0051 (8)	0.0092 (8)
C5	0.0619 (11)	0.0678 (12)	0.0594 (12)	−0.0042 (9)	−0.0057 (9)	0.0031 (9)
C6	0.0797 (14)	0.0726 (13)	0.0498 (11)	0.0064 (11)	−0.0007 (10)	0.0013 (9)
C7	0.0752 (13)	0.0673 (12)	0.0496 (11)	0.0144 (10)	0.0219 (9)	0.0124 (9)
C8	0.0446 (9)	0.0450 (9)	0.0530 (10)	0.0077 (7)	0.0152 (7)	0.0085 (7)
C9	0.0383 (8)	0.0484 (9)	0.0574 (10)	−0.0027 (7)	0.0118 (7)	0.0019 (7)
C10	0.0369 (8)	0.0449 (9)	0.0455 (9)	−0.0026 (6)	0.0119 (7)	0.0005 (7)
C11	0.0366 (8)	0.0479 (9)	0.0443 (9)	−0.0022 (7)	0.0110 (7)	0.0054 (7)
C12	0.0470 (9)	0.0536 (10)	0.0576 (10)	0.0024 (8)	0.0109 (8)	0.0013 (8)
C13	0.0561 (11)	0.0599 (11)	0.0824 (14)	0.0144 (9)	0.0163 (10)	0.0089 (10)
C14	0.0497 (10)	0.0788 (14)	0.0744 (13)	0.0089 (10)	0.0035 (10)	0.0259 (11)
C15	0.0503 (10)	0.0829 (14)	0.0511 (10)	−0.0064 (10)	0.0017 (8)	0.0153 (10)
C16	0.0430 (9)	0.0604 (10)	0.0465 (9)	−0.0070 (8)	0.0110 (7)	0.0066 (8)
C17	0.0434 (9)	0.0565 (10)	0.0567 (10)	−0.0002 (8)	0.0131 (8)	−0.0054 (8)
C18	0.0351 (8)	0.0442 (9)	0.0483 (9)	−0.0013 (6)	0.0108 (7)	0.0011 (7)
C19	0.0574 (11)	0.0509 (11)	0.1275 (18)	0.0076 (9)	0.0519 (12)	0.0222 (11)
C20	0.0654 (12)	0.0509 (11)	0.1321 (19)	0.0138 (9)	0.0516 (13)	0.0227 (11)
C21	0.0400 (9)	0.0618 (11)	0.0593 (10)	0.0086 (8)	0.0163 (8)	0.0024 (8)
C22	0.0508 (10)	0.0704 (12)	0.0821 (13)	0.0072 (9)	0.0362 (9)	0.0218 (10)
C23	0.0503 (10)	0.0539 (10)	0.0723 (12)	0.0079 (8)	0.0264 (9)	0.0198 (9)
C24	0.0460 (10)	0.0738 (12)	0.0634 (12)	0.0099 (9)	0.0171 (8)	0.0044 (9)
N1	0.0533 (9)	0.0929 (13)	0.0860 (12)	0.0226 (9)	0.0265 (8)	0.0099 (9)
N2	0.0539 (9)	0.0728 (10)	0.0486 (9)	−0.0063 (8)	0.0088 (7)	−0.0127 (8)
N3	0.0445 (8)	0.0587 (9)	0.0643 (10)	−0.0013 (7)	0.0253 (7)	0.0109 (7)

### Geometric parameters (Å, °)

**Table:**

C1—C10	1.502 (2)	C12—H12	0.9300
C1—C2	1.503 (2)	C13—C14	1.396 (3)
C1—C18	1.519 (2)	C13—H13	0.9300
C1—H1	0.9800	C14—C15	1.361 (3)
C2—C9	1.356 (2)	C14—H14	0.9300
C2—C3	1.435 (2)	C15—C16	1.384 (2)
C3—C4	1.399 (2)	C15—H15	0.9300
C3—C8	1.402 (2)	C16—N2	1.372 (2)
C4—C5	1.366 (2)	C17—N2	1.368 (2)
C4—H4	0.9300	C17—H17	0.9300
C5—C6	1.389 (3)	C18—C19	1.372 (2)
C5—H5	0.9300	C18—C23	1.375 (2)
C6—C7	1.371 (3)	C19—C20	1.375 (2)
C6—H6	0.9300	C19—H19	0.9300
C7—C8	1.391 (2)	C20—C21	1.373 (2)
C7—H7	0.9300	C20—H20	0.9300
C8—N3	1.369 (2)	C21—C22	1.372 (2)
C9—N3	1.371 (2)	C21—C24	1.437 (2)
C9—H9	0.9300	C22—C23	1.374 (2)
C10—C17	1.358 (2)	C22—H22	0.9300
C10—C11	1.437 (2)	C23—H23	0.9300
C11—C12	1.393 (2)	C24—N1	1.140 (2)
C11—C16	1.404 (2)	N2—H2A	0.91 (2)
C12—C13	1.377 (2)	N3—H3A	0.86 (2)
			
C10—C1—C2	113.60 (12)	C12—C13—H13	119.4
C10—C1—C18	111.50 (12)	C14—C13—H13	119.4
C2—C1—C18	112.65 (12)	C15—C14—C13	121.38 (17)
C10—C1—H1	106.1	C15—C14—H14	119.3
C2—C1—H1	106.1	C13—C14—H14	119.3
C18—C1—H1	106.1	C14—C15—C16	117.70 (17)
C9—C2—C3	106.14 (13)	C14—C15—H15	121.2
C9—C2—C1	128.86 (14)	C16—C15—H15	121.2
C3—C2—C1	124.97 (13)	N2—C16—C15	130.37 (17)
C4—C3—C8	118.91 (15)	N2—C16—C11	107.29 (14)
C4—C3—C2	133.59 (14)	C15—C16—C11	122.30 (17)
C8—C3—C2	107.49 (13)	C10—C17—N2	110.63 (15)
C5—C4—C3	119.26 (16)	C10—C17—H17	124.7
C5—C4—H4	120.4	N2—C17—H17	124.7
C3—C4—H4	120.4	C19—C18—C23	117.96 (14)
C4—C5—C6	121.14 (18)	C19—C18—C1	121.60 (13)
C4—C5—H5	119.4	C23—C18—C1	120.43 (14)
C6—C5—H5	119.4	C18—C19—C20	121.58 (16)
C7—C6—C5	121.16 (18)	C18—C19—H19	119.2
C7—C6—H6	119.4	C20—C19—H19	119.2
C5—C6—H6	119.4	C21—C20—C19	119.69 (17)
C6—C7—C8	118.03 (17)	C21—C20—H20	120.2
C6—C7—H7	121.0	C19—C20—H20	120.2
C8—C7—H7	121.0	C22—C21—C20	119.55 (15)
N3—C8—C7	131.44 (16)	C22—C21—C24	120.41 (16)
N3—C8—C3	107.05 (14)	C20—C21—C24	120.04 (16)
C7—C8—C3	121.50 (16)	C21—C22—C23	120.03 (15)
C2—C9—N3	110.08 (15)	C21—C22—H22	120.0
C2—C9—H9	125.0	C23—C22—H22	120.0
N3—C9—H9	125.0	C22—C23—C18	121.19 (16)
C17—C10—C11	105.91 (14)	C22—C23—H23	119.4
C17—C10—C1	128.49 (14)	C18—C23—H23	119.4
C11—C10—C1	125.50 (13)	N1—C24—C21	178.7 (2)
C12—C11—C16	118.77 (14)	C17—N2—C16	108.82 (14)
C12—C11—C10	133.90 (15)	C17—N2—H2A	125.3 (14)
C16—C11—C10	107.31 (14)	C16—N2—H2A	125.7 (14)
C13—C12—C11	118.70 (17)	C8—N3—C9	109.24 (13)
C13—C12—H12	120.7	C8—N3—H3A	126.3 (13)
C11—C12—H12	120.7	C9—N3—H3A	124.4 (13)
C12—C13—C14	121.11 (18)		
			
C10—C1—C2—C9	−10.3 (2)	C11—C12—C13—C14	−0.5 (3)
C18—C1—C2—C9	117.73 (17)	C12—C13—C14—C15	−0.8 (3)
C10—C1—C2—C3	167.30 (13)	C13—C14—C15—C16	0.5 (3)
C18—C1—C2—C3	−64.69 (18)	C14—C15—C16—N2	178.63 (17)
C9—C2—C3—C4	179.30 (17)	C14—C15—C16—C11	1.1 (2)
C1—C2—C3—C4	1.3 (3)	C12—C11—C16—N2	179.62 (14)
C9—C2—C3—C8	0.43 (16)	C10—C11—C16—N2	−1.61 (17)
C1—C2—C3—C8	−177.61 (13)	C12—C11—C16—C15	−2.3 (2)
C8—C3—C4—C5	0.9 (2)	C10—C11—C16—C15	176.45 (14)
C2—C3—C4—C5	−177.91 (16)	C11—C10—C17—N2	−0.21 (18)
C3—C4—C5—C6	−0.9 (3)	C1—C10—C17—N2	176.26 (14)
C4—C5—C6—C7	0.6 (3)	C10—C1—C18—C19	87.1 (2)
C5—C6—C7—C8	−0.3 (3)	C2—C1—C18—C19	−42.0 (2)
C6—C7—C8—N3	179.15 (17)	C10—C1—C18—C23	−91.44 (18)
C6—C7—C8—C3	0.3 (3)	C2—C1—C18—C23	139.46 (16)
C4—C3—C8—N3	−179.68 (14)	C23—C18—C19—C20	0.7 (3)
C2—C3—C8—N3	−0.62 (17)	C1—C18—C19—C20	−177.84 (19)
C4—C3—C8—C7	−0.6 (2)	C18—C19—C20—C21	−1.1 (4)
C2—C3—C8—C7	178.47 (14)	C19—C20—C21—C22	0.6 (3)
C3—C2—C9—N3	−0.08 (17)	C19—C20—C21—C24	−178.70 (19)
C1—C2—C9—N3	177.86 (14)	C20—C21—C22—C23	0.2 (3)
C2—C1—C10—C17	115.39 (17)	C24—C21—C22—C23	179.56 (18)
C18—C1—C10—C17	−13.2 (2)	C21—C22—C23—C18	−0.6 (3)
C2—C1—C10—C11	−68.78 (18)	C19—C18—C23—C22	0.2 (3)
C18—C1—C10—C11	162.63 (13)	C1—C18—C23—C22	178.74 (16)
C17—C10—C11—C12	179.63 (17)	C10—C17—N2—C16	−0.81 (19)
C1—C10—C11—C12	3.0 (3)	C15—C16—N2—C17	−176.35 (17)
C17—C10—C11—C16	1.12 (17)	C11—C16—N2—C17	1.50 (18)
C1—C10—C11—C16	−175.49 (14)	C7—C8—N3—C9	−178.39 (17)
C16—C11—C12—C13	2.0 (2)	C3—C8—N3—C9	0.58 (17)
C10—C11—C12—C13	−176.41 (16)	C2—C9—N3—C8	−0.32 (18)

### Hydrogen-bond geometry (Å, °)

**Table:**

*D*—H···*A*	*D*—H	H···*A*	*D*···*A*	*D*—H···*A*
N3—H3A···N1^i^	0.86 (2)	2.22 (2)	3.084 (2)	178.6 (18)
N2—H2A···N1^ii^	0.91 (2)	2.34 (2)	3.206 (2)	160.3 (19)

Symmetry codes: (i) *x*+1, −*y*+1/2, *z*−1/2; (ii) *x*+1, −*y*+1/2, *z*+1/2.

## References

[bb1] Allen, F. H., Kennard, O., Watson, D. G., Brammer, L., Orpen, A. G. & Taylor, R. (1987). *J. Chem. Soc. Perkin Trans. 2*, pp. S1–19.

[bb2] Bell, R., Carmeli, S., Sar, N. & Vibrindole, A. (1994). *J. Nat. Prod.* **57**, 1587–1590. 10.1021/np50113a022 7853008

[bb3] Bruker (2007). *SMART* and *SAINT* Bruker AXS Inc., Madison, Wisconsin, USA.

[bb4] Govindasamy, L., Velmurugan, D., Ravikumar, K. & Mohanakrishnan, A. K. (1998). *Acta Cryst.* C**54**, 635–637.

[bb5] Krishna, R., Velmurugan, D., Babu, G. & Perumal, P. T. (1999). *Acta Cryst.* C**55**, 75–78.

[bb6] Krishna, R., Velmurugan, D. & Shanmuga Sundara, S. (1999). *Acta Cryst.* C**55**, IUC9900084.

[bb7] Seetharaman, J. & Rajan, S. S. (1995). *Acta Cryst.* C**51**, 78–80.

[bb8] Sheldrick, G. M. (2008). *Acta Cryst.* A**64**, 112–122. 10.1107/S0108767307043930 18156677

[bb9] Spek, A. L. (2009). *Acta Cryst.* D**65**, 148–155. 10.1107/S090744490804362X PMC2631630 19171970

